# Enhancing Clinical Documentation and Management of Cauda Equina Syndrome: The Development and Impact of a Structured Assessment Proforma

**DOI:** 10.7759/cureus.86226

**Published:** 2025-06-17

**Authors:** Omar Al-Doori, Mustafa Al-Jaafar

**Affiliations:** 1 Orthopaedics and Trauma, Aneurin Bevan University Health Board, Wales, GBR; 2 Trauma and Orthopaedics, The Grange University Hospital, Cwmbran, GBR

**Keywords:** cauda equina syndrome, clinical assessment, documentation, medico-legal, proforma

## Abstract

Background

Cauda equina syndrome (CES) is a neurosurgical emergency associated with potentially devastating neurological sequelae and significant medico-legal implications. Timely surgical decompression is critical; however, effective management relies on precise documentation of clinical findings. In many settings, documentation of key neurovascular assessments remains suboptimal. This study aimed to evaluate and improve the quality of clinical documentation in suspected CES cases by implementing a structured assessment proforma, thereby facilitating timely and accurate clinical evaluation, expediting neurosurgical referral, and mitigating legal risks.

Methodology

A retrospective review was conducted to compare documentation practices in 50 patients with suspected CES before and 50 patients after the introduction of a custom-designed proforma in the Trauma and Orthopaedic Department. Documentation was evaluated for the following nine key parameters: clinical history, motor examination, sensory examination, bladder function, bowel function, reflexes, perianal pinprick test, sexual dysfunction assessment, and radicular pain. Referral times for urgent surgical intervention were also recorded.

Results

Implementation of the proforma significantly improved documentation rates. Perianal pinprick testing increased from 12 (24%) cases pre-proforma to 44 (88%) cases post-proforma. Bladder function documentation improved from 31 (62%) cases to 49 (98%) cases, and sexual history documentation increased from six (12%) cases to 46 (92%) cases. Among patients requiring urgent surgical intervention, the time from referral to definitive management was reduced by an estimated seven hours. Eight urgent cases were identified pre-proforma, compared to five post-proforma.

Conclusions

The structured assessment proforma significantly enhanced the completeness of neurovascular documentation and streamlined the management of suspected CES cases, resulting in a notable reduction in referral delays for urgent intervention. Adoption of such a proforma may improve diagnostic accuracy, expedite neurosurgical referrals, and reduce medico-legal risk.

## Introduction

The term “cauda equina,” derived from the Latin for “horsetail,” was first coined by French anatomist André du Laurens to describe the bundle of lumbar, sacral, and coccygeal nerve roots surrounding the filum terminale [1]. Cauda equina syndrome (CES) is a rare but critical neurological emergency characterized by compression of these nerve roots, most commonly due to a herniated intervertebral disc [2]. This compression can lead to profound dysfunction, including loss of bladder, bowel, and sexual functions, and necessitates prompt surgical decompression to avert permanent neurological sequelae [3].

Recent classifications of CES recognize distinct clinical stages: suspected CES, in which patients exhibit either bilateral sciatica or sensory deficits without sphincteric symptoms; symptom-only CES, marked by perineal sensory loss despite intact bladder, bowel, and sexual function; incomplete CES, featuring altered bladder sensation or function with preserved voiding capacity; CES with retention, in which painless urinary retention and overflow occur; and complete CES, characterized by total loss of bladder control, perineal sensation, and anal tone [4]. Accurate and timely diagnosis is challenging, as CES may only be recognized after significant neurological compromise has occurred [5]. Although no universally accepted definition exists, CES is broadly defined by a combination of sensory and motor deficits alongside bowel and bladder dysfunction [6].

A comprehensive clinical evaluation, often supplemented by MRI or, where contraindicated, CT myelography, is essential for diagnosis. Upon suspicion of CES, urgent referral for neurosurgical assessment and decompression is indicated to optimize outcomes and mitigate medico-legal risk [5]. Indeed, the medico-legal ramifications of CES are disproportionately high relative to its prevalence, largely due to the severe consequences of delayed or missed diagnosis [7]. Inadequate documentation of key clinical findings, i.e., motor and sensory assessments, sphincteric function, reflex testing, and perianal sensation, can significantly contribute to diagnostic delays, compromising patient care and increasing litigation risk [3].

Despite consensus on the importance of thorough documentation, practice varies widely, and assessment records are often incomplete [8]. To address these gaps, we developed a structured assessment proforma aimed at standardizing documentation, ensuring comprehensive recording of critical clinical parameters, and facilitating timely surgical intervention.

## Materials and methods

Study design and setting

This retrospective, before-and-after audit was conducted at The Grange University Hospital, under the Aneurin Bevan University Health Board, from May 1 to September 30, 2023. The primary aim was to evaluate the completeness of clinical documentation, thereby mitigating medico-legal risk and facilitating timely surgical management, rather than directly measuring patient outcomes.

Patient selection

All adult patients (≥18 years) referred to the Orthopaedic Department as suspected CES from the Emergency Department, physiotherapy services, or primary care (General Practitioners) were eligible. Each referral required documented lower back pain plus one or more red-flag symptoms (e.g., new-onset saddle anesthesia, urinary retention, fecal incontinence) prompting an accident and emergency review.

Inclusion Criteria

Referral for suspected CES as defined above. Initial assessment performed by the Accident and Emergency or Orthopaedic team.

Exclusion Criteria

Presenting complaints or imaging confirming an alternative diagnosis (e.g., spinal tumor, peripheral neuropathy) that fully explained symptoms. Any record lacking a CES-focused assessment (i.e., no mention of red-flag symptoms or neurological examination).

In total, 50 consecutive eligible patients assessed before proforma introduction (May 1 to June 30, 2023) comprised the pre-proforma group; 50 consecutive patients after proforma introduction (July 1 to August 31, 2023) comprised the post-proforma group. A second audit of the post-proforma documentation was conducted in September 2023 to evaluate sustained adherence.

Statistical analysis

Descriptive statistics were used to summarize categorical data, expressed as frequencies and percentages. The effectiveness of the structured documentation proforma was assessed by comparing pre- and post-intervention documentation rates using 2 × 2 contingency tables. Fisher’s exact test was used when expected cell counts were fewer than five, and chi-square tests were used when all assumptions were met. For paired categorical comparisons within the same cohort (e.g., measuring MRI reporting time from two different starting points), McNemar’s test was employed. A p-value <0.05 was considered statistically significant. All analyses were performed using validated online statistical tools (GraphPad QuickCalcs, GraphPad Software, San Diego, CA, USA).

Data collection

Data were collected from the Trauma and Orthopaedic Department post‑take list and retrospectively extracted from the hospital clinical workstation. Using a standardized data dictionary, trained registrars extracted the following information from patient records: (1) clinical history completeness (all red flags documented); (2) motor examination (specific myotome grading 0-5); (3) sensory examination (specific dermatome pinprick/light touch); (4) bladder function (voiding status with retention volume or incontinence onset); (5) bowel function (noted episodes of incontinence or constipation); (6) deep tendon reflexes (knee and ankle graded); (7) perianal pinprick sensation; (8) sexual dysfunction (presence and type); (9) radicular pain (presence/absence); (10) timing metrics: time of first assessment, MRI request, MRI report, and definitive surgical intervention for confirmed CES cases. Any examination element documented without the required detail (e.g., “motor deficit noted” without myotome level) was classified as not documented.

Proforma implementation

In June 2023, a structured assessment proforma was introduced. The double-sided form included sections for patient identifiers and time/clinician of first assessment, red-flag checklist, neurological exam fields with mandatory sub-fields (e.g., specific myotome for motor deficits, dermatome for sensory loss), documentation of bladder dysfunction (retention vs. incontinence with time frame), and MRI request and report timestamps. Registrars/Senior house officers received brief training on its use (Appendices).

## Results

Demographics

The median age was 39 years. Of the 100 patients, 80 (80%) were female and 20 (20%) were male.

Documentation quality

The implementation of the structured clinical documentation proforma resulted in significant improvements across all domains of neurological and clinical assessments.

Motor function documentation increased from 80% (40 out of 50 cases) before the intervention to 96% (48 out of 50 cases) after the intervention. This improvement was statistically significant, as confirmed by Fisher’s exact test (p = 0.0277). Sensory function documentation also improved markedly from 78% (39 out of 50 cases) to 96% (48 out of 50 cases), with a statistically significant difference noted (Fisher’s exact test, p = 0.0147).

Similarly, reflex assessment documentation increased from 62% (31 out of 50 cases) to 96% (48 out of 50 cases), a highly significant improvement (Fisher’s exact test, p < 0.001). Documentation of perianal pinprick testing, a critical component in evaluating suspected CES, showed the most substantial improvement, rising from 24% (12 out of 50 cases) to 88% (44 out of 50 cases). This increase was statistically significant based on the chi-square test (χ² = 39.0, p < 0.001).

Bladder function documentation improved from 62% (31 out of 50 cases) to 98% (49 out of 50 cases), achieving statistical significance using Fisher’s exact test (p < 0.001). Bowel function documentation also improved significantly from 66% (33 out of 50 cases) to 90% (45 out of 50 cases) (Fisher’s exact test, p < 0.001).

Bladder scan documentation increased from 60% (30 out of 50 cases) to 94% (47 out of 50 cases), with Fisher’s exact test confirming this as a significant improvement (p < 0.001). Notably, documentation of sexual history, a historically underreported element, improved dramatically from 12% (6 out of 50 cases) to 92% (46 out of 50 cases). This increase was statistically significant with the chi-square test indicating significance (χ² = 8.38, p = 0.0038).

**Figure 1:**
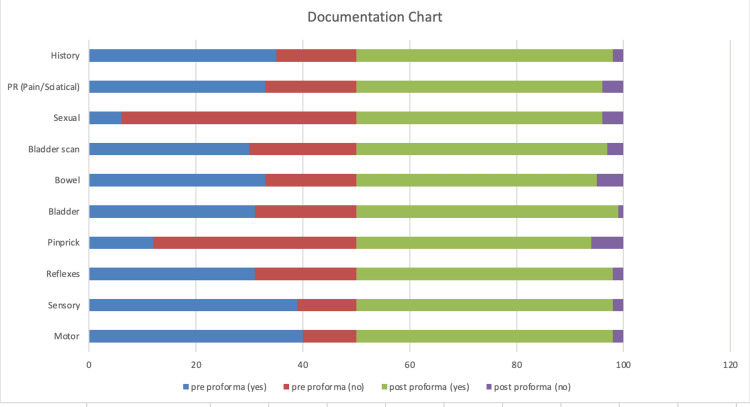
Documentation chart.

Pain or sciatica documentation improved from 66% (33 out of 50 cases) to 92% (46 out of 50 cases), and this change was statistically significant (Fisher’s exact test, p = 0.0033). Overall, the completeness of history documentation increased from 70% (35 out of 50 cases) to 96% (48 out of 50 cases), with Fisher’s exact test confirming the significance of this improvement (p = 0.001). Further details are provided in Table 1 and Figure 1.

**Table 1:** Improvements in clinical documentation following proforma implementation. Fisher’s exact test was used for comparisons where expected cell counts were low. The chi-square test (χ²) was used when assumptions for the chi-square test were met.

Clinical assessment	Pre-intervention, Yes (%)	Post-intervention, Yes (%)	Statistical test	Test statistic	P-value
Motor function	40/50 (80%)	48/50 (96%)	Fisher’s exact	–	0.0277
Sensory function	39/50 (78%)	48/50 (96%)	Fisher’s exact	–	0.0147
Reflexes	31/50 (62%)	48/50 (96%)	Fisher’s exact	–	<0.001
Perianal pinprick	12/50 (24%)	44/50 (88%)	Chi-square	χ² = 39.0	<0.001
Bladder function	31/50 (62%)	49/50 (98%)	Fisher’s exact	–	<0.001
Bowel function	33/50 (66%)	45/50 (90%)	Fisher’s exact	_	<0.001
Bladder scan	30/50 (60%)	47/50 (94%)	Fisher’s exact	–	<0.001
Sexual history	6/50 (12%)	46/50 (92%)	Chi-square	χ² = 8.38	0.0038
Pain/Sciatica	33/50 (66%)	46/50 (92%)	Fisher’s exact	–	0.0033
Overall history	35/50 (70%)	48/50 (96%)	Fisher’s exact	–	0.001

MRI and management timing

Improvements were also noted in the efficiency of MRI reporting and overall patient management following the implementation of the structured proforma. When comparing time to MRI reporting, a significant increase was observed in the proportion of patients receiving MRI results within four hours. Specifically, when measured from the point of initial clinical review, 44% of patients (22 out of 50) met this threshold, compared to 70% (35 out of 50) when timing was calculated from the orthopedic doctor’s formal MRI request. This increase was statistically significant, as confirmed by McNemar’s test (p < 0.001), suggesting a more streamlined and consistent imaging pathway.

## Discussion

CES is most frequently precipitated by a prolapsed intervertebral disc, a condition that can result in profound neurological disability. Mismanagement of CES not only jeopardizes patient outcomes but also exposes healthcare providers to substantial medico-legal risk [3,9]. Gardner et al. reported that inadequate or delayed management of CES is often associated with high litigation costs [3], while Hussain et al. found that poor outcomes in CES have far-reaching implications for both patient quality of life and the healthcare system [9]. Markham highlighted that delays in diagnosis and treatment markedly increase litigation risk, reinforcing the critical importance of timely management and comprehensive patient communication [10].

The clinical presentation of CES is notably heterogeneous. Tandon and Sankaran described three distinct variations in its presentation, namely, a rapid onset form in patients without a prior history of back problems suggestive of an acute and severe disc herniation; acute bladder dysfunction in individuals with a background of low back pain and sciatica reflecting an evolution from chronic degenerative changes; and chronic backache and sciatica with gradual progression to CES, often in association with spinal canal stenosis [11]. This classification underscores the variable clinical manifestations of CES and reinforces the need for standardized diagnostic protocols that facilitate early recognition and timely intervention.

In emergency departments, rapid intervention is critical for the effective management of CES. Ideally, a dedicated CES protocol should be implemented to ensure that the clinical diagnosis is promptly verified and that the spinal surgery team is immediately notified. Baseline neurological assessments, including evaluations of anal sphincter tone and perianal pinprick sensation, must be systematically recorded to facilitate early detection and timely intervention [12]. The utilization of a bladder scanner can significantly aid in assessing bladder function, and the results of such scans should be diligently documented as part of the overall clinical evaluation [13,14].

Early surgical intervention has been consistently associated with improved urological outcomes in patients with CES. Shapiro and Robinson documented urological recovery rates ranging from 70% to 100% when treatment was initiated within 48 hours of symptom onset [15,16]. Conversely, delayed intervention beyond 48 hours is associated with persistent and significant sphincter dysfunction, as demonstrated by Jennett and Nielsen et al. [17,18]. This body of evidence underscores the critical importance of timely diagnosis and prompt decompression to optimize both functional recovery and overall prognosis in CES.

Outcomes in CES are strongly determined by both the timing and severity of the initial neural injury. Kennedy et al. demonstrated that patients presenting with extensive neurological deficits, particularly when there is a delay in surgical decompression, tend to have a significantly poorer prognosis, often resulting in persistent functional impairments despite intervention [19]. Even when treatment is initiated promptly, patients with CES may continue to experience significant sphincter and lower limb deficits, leading to considerable disability and dependency. This residual impairment frequently prompts patients and their legal representatives to pursue claims of negligence. Rather than engaging in a costly, emotionally taxing, and ultimately unproductive legal process, patients may benefit more from accepting their condition and focusing on mitigating its impact through comprehensive medical rehabilitation and robust social support [3].

Delays and mismanagement in the diagnosis and treatment of CES open the door for substantial litigation related to long-term disabilities [20]. Data from the NHS Litigation Authority (NHSLA) indicate that, despite its rarity, CES significantly contributes to medico-legal costs. Between April 1, 2003, and March 31, 2008, the NHSLA received 78 CES-related claims, with resolved cases yielding an average compensation of £211,758 per case, amounting to a total of £2,541,098 over five years, or approximately £508,219 annually. The largest settlement reached £2,041,000, and of the 78 claims, 39 were linked to orthopedics, 21 to accident and emergency, seven to neurology or neurosurgery, and 11 to other departments [21].

Limitations

A limitation of this study is its retrospective, single-center design with a relatively small sample size, which may limit generalizability. In addition, because documentation was reviewed from medical records, there is potential for observer bias in how thoroughly clinicians completed the proforma versus free-text notes. Finally, we did not capture long-term functional or legal outcomes, which future prospective studies should address.

## Conclusions

The introduction of a structured assessment proforma in the evaluation of suspected CES fostered a more systematic and comprehensive approach to clinical examination. By standardizing critical assessment steps, motor and sensory testing, sphincteric evaluation, and documentation of red-flag symptoms, this tool enhanced communication among multidisciplinary teams and streamlined decision-making. Moreover, clearer recording of MRI request and report times ensured more efficient imaging workflows and expedited specialist referrals. Taken together, these improvements have the potential to elevate diagnostic accuracy, shorten delays to definitive management, and reduce the risk of adverse outcomes and medico-legal exposure. We recommend that other centers adopt similar structured protocols and conduct prospective studies to assess their effects on functional recovery, patient satisfaction, resource utilization, and litigation rates.

## Figures and Tables

**Figure 2:**
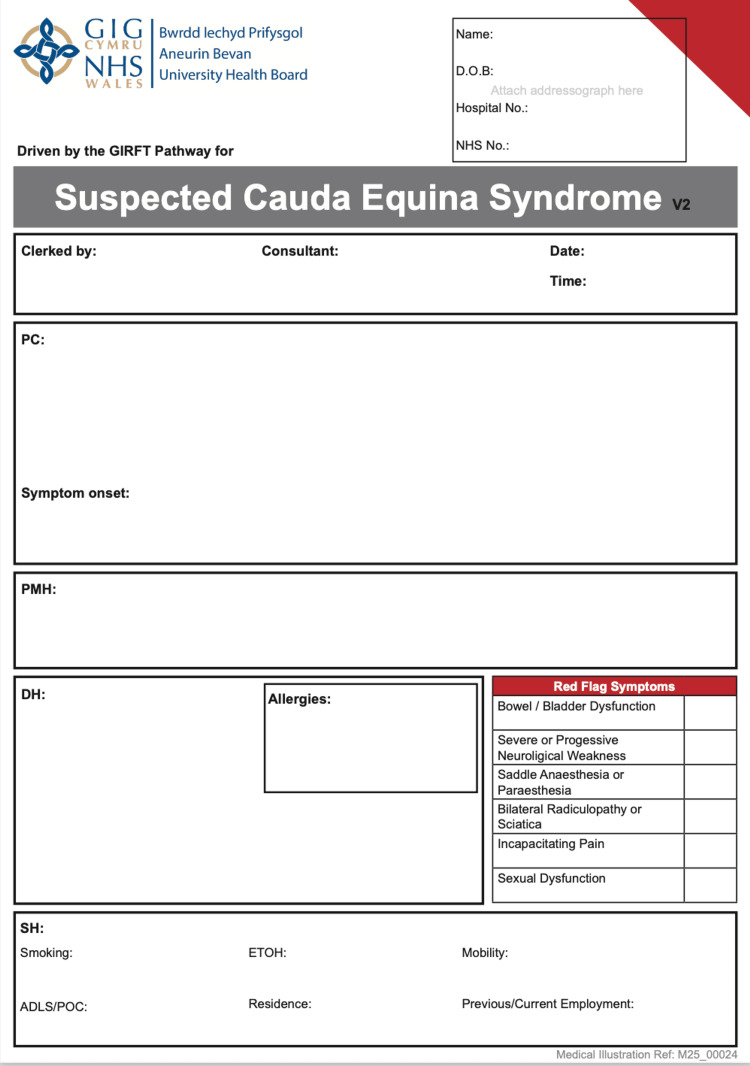
Front page of the proforma.

**Figure 3:**
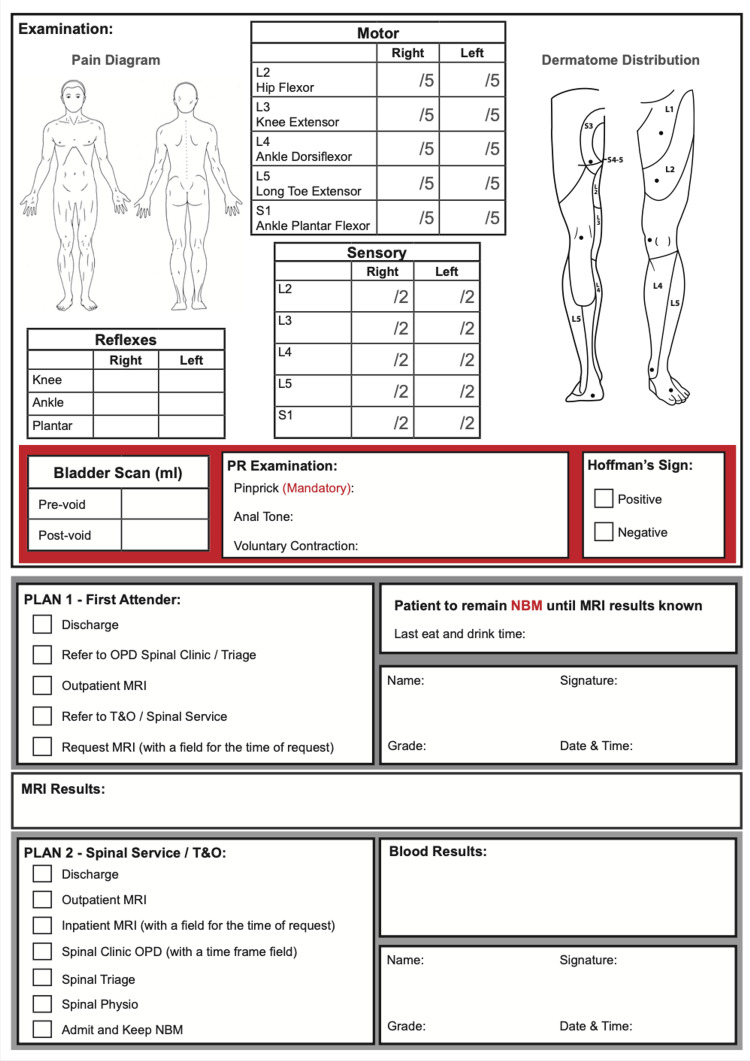
Back page of the proforma.

## References

[1] Olry R, Haines DE (2012). Between André Du Laurens' horse tail and William Cadogan's pony tail. J Hist Neurosci.

[2] Kapetanakis S, Chaniotakis C, Kazakos C, Papathanasiou JV (2017). Cauda equina syndrome due to lumbar disc herniation: a review of literature. Folia Med (Plovdiv).

[3] Gardner A, Gardner E, Morley T (2011). Cauda equina syndrome: a review of the current clinical and medico-legal position. Eur Spine J.

[4] Lavy C, Marks P, Dangas K, Todd N (2022). Cauda equina syndrome-a practical guide to definition and classification. Int Orthop.

[5] Kuris EO, McDonald CL, Palumbo MA, Daniels AH (2021). Evaluation and management of cauda equina syndrome. Am J Med.

[6] Fraser S, Roberts L, Murphy E (2009). Cauda equina syndrome: a literature review of its definition and clinical presentation. Arch Phys Med Rehabil.

[7] Kostuik JP (2004). Medicolegal consequences of cauda equina syndrome: an overview. Neurosurg Focus.

[8] Mehta N, Garbera D, Kaye J, Ramakrishnan M (2015). Documentation of focal neurology on patients with suspected cauda equina syndrome and the development of an assessment proforma. Open Orthop J.

[9] Hussain SA, Gullan RW, Chitnavis BP (2003). Cauda equina syndrome: outcome and implications for management. Br J Neurosurg.

[10] Markham D (2004). Cauda equina syndrome: diagnosis, delay and litigation risk. Curr Orthop.

[11] Tandon P, Sankaran B (1967). Cauda equina syndrome due to lumbar disc prolapse. Indian J Orthop.

[12] Todd NV (2005). Cauda equina syndrome: the timing of surgery probably does influence outcome. Br J Neurosurg.

[13] Zhong J, Jia S, Pu F, Niu H, Li S, Li D, Fan Y (2010). Ultrasound estimation of female bladder volume based on magnetic resonance modeling. J Urol.

[14] Venkatesan M, Nasto L, Tsegaye M, Grevitt M (2019). Bladder scans and postvoid residual volume measurement improve diagnostic accuracy of cauda equina syndrome. Spine (Phila Pa 1976).

[15] Shapiro S (1993). Cauda equina syndrome secondary to lumbar disc herniation. Neurosurgery.

[16] Robinson RG (1965). Massive protrusions of lumbar disks. Br J Surg.

[17] Jennett WB (1956). A study of 25 cases of compression of the cauda equina by prolapsed intervertebral discs. J Neurol Neurosurg Psychiatry.

[18] Nielsen B, de Nully M, Schmidt K, Hansen RI (1980). A urodynamic study of cauda equina syndrome due to lumbar disc herniation. Urol Int.

[19] Kennedy JG, Soffe KE, McGrath A, Stephens MM, Walsh MG, McManus F (1999). Predictors of outcome in cauda equina syndrome. Eur Spine J.

[20] Gleave JR, Macfarlane R (2002). Cauda equina syndrome: what is the relationship between timing of surgery and outcome?. Br J Neurosurg.

[21] Symons R (2008). NHS Litigation Authority Data on CES-Related Claims.

